# Calcaneus height is a key morphological factor of sprint performance in sprinters

**DOI:** 10.1038/s41598-020-72388-7

**Published:** 2020-09-22

**Authors:** Tadashi Suga, Msafumi Terada, Takahiro Tanaka, Yuto Miyake, Hiromasa Ueno, Mitsuo Otsuka, Akinori Nagano, Tadao Isaka

**Affiliations:** 1 Faculty of Sport and Health Science, Ritsumeikan University, 1-1-1 Nojihigashi, Kusatsu, Shiga 525-8577 Japan; 2 Graduate School of Health and Sport Science, Nippon Sport Science University, Tokyo, Japan; 3 Japan Society for the Promotion of Science, Tokyo, Japan

**Keywords:** Physiology, Anatomy

## Abstract

This study examined the relationships between the foot bone morphologies and sprint performance in sprinters. Foot images in 56 male sprinters obtained using magnetic resonance imaging. The relative lengths of the forefoot bones of the big and second toes, which were calculated as total lengths of the forefoot bones for each toe normalized to the foot length, correlated significantly with personal best 100-m sprint time (*r* =  − 0.293 and − 0.459, both *P*s < 0.05). The relative lengths of the rearfoot talus and calcaneus normalized to the foot length also correlated significantly with the sprint performance (*r* =  − 0.378 and − 0.496, both *P*s < 0.05). Furthermore, the relative height of the calcaneus, but not the talus, normalized to body height correlated significantly with sprint performance (*r* =  − 0.690, *P* < 0.001). Additionally, the relative calcaneus height correlated significantly with the foot arch height index (*r* = 0.420, *P* = 0.001), and the foot arch height index correlated significantly with sprint performance (*r* =  − 0.517, *P* < 0.001). These findings suggest that the taller calcaneus may be a key morphological factor for achieving superior sprint performance, potentially via modeling the longer forefoot and rearfoot bones and functional foot morphology in sprinters.

## Introduction

Superior sprint performance is achieved using gross torques of the lower limb joints, including the hip, knee, and ankle. Of these components, greater ankle plantar flexor torque plays an important role in increasing ground reaction forces and shortening contact time during the stance phases while sprinting^1^, which are the kinematic and kinetic determinants of sprint performance^2^. Dowson et al.^3^ reported that greater plantar flexor isokinetic torque correlated with superior sprint performance in athletes, including sprinters. In general, the magnitude of joint torque is primarily determined by the agonist muscle size^4,5^; therefore, greater plantar flexor muscle may be related to superior sprint performance in sprinters. Despite the fact that the relationship between the plantar flexor muscle size and sprint performance in sprinters has been inconsistently remained among the results of previous studies^6–10^, we and others reported the absence of this relationship^6,9,10^. Therefore, sprinters may have other morphologies than muscle size related to increase in plantar flexor torque while sprinting.

We and others previously demonstrated that the forefoot bones were longer in sprinters than in non-sprinters^11–14^. This result suggests that the longer forefoot bones may be an essential characteristic for successful sprinters. Furthermore, using computer simulation, Lee and Piazza^12^ determined that a longer forefoot contributes to the production of greater plantar flexor torque during the push-off phase while sprinting. Given their findings, we demonstrated that longer forefoot bones correlated with better sprint performance in sprinters^13,14^. Therefore, the forefoot bone length is an important morphological factor of sprint performance in sprinters.

When having the longer forefoot bones, shorter rearfoot bones may be required for modeling the foot, because of a balance between lengths of the forefoot and rearfoot bones. In fact, Baxter et al.^11^ determined that the plantar flexor moment arm (MA) dimension (i.e., the rearfoot length) was shorter in sprinters than in non-sprinters. Raichlen et al.^15^ reported a positive correlation between the plantar flexor MA and the calcaneus length. Considering these findings, shorter rearfoot bones, especially the calcaneus, may be a required characteristic for achieving superior sprinter performance in sprinters. Nevertheless, Baxter and Piazza^16^ determined that greater plantar flexor MA correlated with higher plantar flexor isometric and isokinetic torques. Their result is consistent with the results of the knee joint in previous studies^17,18^. Because of the close relationship between plantar flexor torque and sprint performance^3^, a shorter calcaneus may be a disadvantage in producing greater plantar flexor torque and achieving superior sprint performance. Therefore, the first hypothesis of the present study was that longer, rather than shorter, rearfoot bones, especially the calcaneus, would be related to better sprint performance in sprinters.

We previously reported that, although the lengths of the forefoot bones were longer in sprinters than in non-sprinters, the lengths of the mid-foot bones (i.e., medial cuneiform, intermediate cuneiform, and navicular) did not differ between the two groups; therefore, we concluded that the mid-foot bones do not display specific modeling for sprinters^13,14^. Based on the results of our previous studies, the second hypothesis of the present study was that, when having a taller rearfoot bones, especially the calcaneus, it would be required for modeling longer forefoot and rearfoot bones and would be related to better sprint performance in sprinters.

Previous studies reported that the foot arch height may help utilize the elastic energy stored by the metatarsophalangeal (MTP) joint during the stance phase while sprinting^19–21^. Hence, the foot arch height may play an important role in producing higher plantar flexor torque because of increased MTP joint torque using the stored energy during push-off in the stance phase^11,12,19–21^. Morita et al.^22^ reported that the foot arch height correlated with 50-m sprint time in children. Despite these findings, to the best of our knowledge, no study has examined the relationship between foot arch height and sprint performance in adults, including sprinters. The foot arch height is often associated with the foot length; however, this relationship is inconsistent among the findings of the previous studies^23,24^. Nevertheless, a study by Morita et al.^22^ did not find a correlation between foot length and sprint performance in children. Furthermore, several previous studies reported that the foot length did not differ between faster and slower groups of adult sprinters^11,25^. Based on these previous findings, the foot arch height may relate to sprint performance independently of the foot length; in other words, the foot arch height may be a specific morphology for determining sprint performance among the foot characteristics. If there is a correlation between foot arch height and sprint performance in adult sprinters, it may be due to a taller calcaneus, which could contribute to modeling a higher foot arch height, potentially because of increased navicular height. Therefore, the third hypothesis of the present study was that taller calcaneus would be related to higher foot arch height, and that the higher foot arch height would be related to better sprint performance in sprinters.

To test these hypotheses, we first compared the lengths and heights of the forefoot and rearfoot bones and foot arch height between faster and slower sprinters who had been divided based on their personal best times in a 100-m sprint race, which is due to understand the foot morphological characteristics in sprinters having superior sprint performance. Thereafter, we examined the relationship between these foot morphologies and sprint performance in sprinters.

## Methods

### Participants

Fifty-six well-trained male sprinters (age, 20.8 ± 1.8 years) participated in this study. All sprinters were involved in regular sprint training and competition. Their best personal times in a 100-m sprint ranged from 10.21 to 11.90 s (mean, 11.10 ± 0.41 s). In order to understand the foot characteristics in sprinters having superior sprint performance, sprinters were divided into two groups, one consisting of faster sprinters (n = 28) and one consisting of slower groups (n = 28), based on the personal best 100-m sprint time, as in our previous study^13^. The personal best 100-m sprint time of faster sprinters ranged from 10.21 to 11.18 s (mean, 10.79 ± 0.30 s). The personal best 100-m sprint time of slower sprinters ranged from 11.19 to 11.90 s (mean, 11.42 ± 0.20 s). The participants were informed of the experimental procedures and provided written consent to participate in the study. None of the participants had contraindications to magnetic resonance imaging. All participants were informed of the experimental procedures and provided written consent to participate in the study. The study was approved by the Ethics Committee of Ritsumeikan University (BKC-IRB-2016-047) and conducted according to the Declaration of Helsinki.

### Foot anthropometric parameters

Length of the right foot of participants was measured in millimeters as the maximum distance from the heel to the end of the big and second toes. The highest value in both toes was considered the maximum foot length, and this length was used to normalize the lengths of the forefoot and rearfoot bones.

### Magnetic resonance imaging

The scans of the right foot of participants were performed using a 1.5-T magnetic resonance system (Signa HDxt; GE Medical Systems, USA). The participants were positioned supine on the scanner bed, with both knees fully extended. The subject’s right ankle was carefully set at a neutral position (i.e., 0º). The foot scans were acquired using a 4-channel foot ankle coil. Three-dimensional isotopic T1-weighted images were obtained with a repetition time of 11.3 s, echo time of 5.1 ms, slice thickness of 1.2 mm, field of view of 28 cm, and matrix size of 256 × 256 pixels. The analyses of the foot morphological variables were conducted using image analysis software (OsiriX Version 5.6; OsiriX Foundation, Switzerland). All foot morphological variables were measured twice by a single examiner, and the two values were averaged.

### Foot bone morphological variables

**Figure 1:**
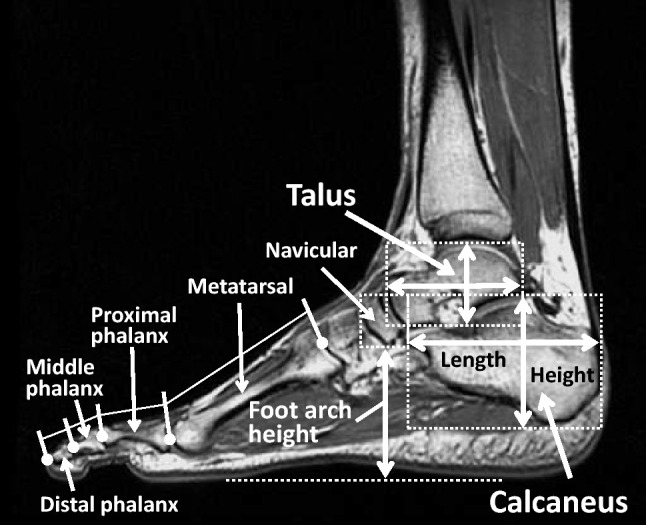
Representative magnetic resonance image for measuring morphological variables of the foot. The lengths of the forefoot bones of the big (included the distal phalanx, proximal phalanx, and metatarsal) and second (included the distal phalanx, middle phalanx, proximal phalanx, and metatarsal) toes were measured along the long axis of the bone between the intersections of the long axis and the cortex at the distal and proximal ends and then totaled to assess overall forefoot bone length for each toe. The lengths and heights of the talus and calcaneus were measured as maximal distances between the cortexes at the distal and proximal ends. The foot arch height was measured as the minimum vertical distance between the navicular and the line connecting the thenar eminence to the bottom of the heel.

Representative magnetic resonance imaging scans for measuring the foot morphological variables bones are shown in Fig. 1. The forefoot bones of the big toe included the distal phalanx, proximal phalanx, and metatarsal. The forefoot bones of the second toe included the distal phalanx, middle phalanx, proximal phalanx, and metatarsal. These bone lengths located at each toes were measured along the long axis of the bone between the intersections of the long axis and the cortex at the distal and proximal ends^13,14^. The coefficient of variations of the two measurements for the forefoot bone lengths of the big and second toes in all participants were: 1.6 ± 1.2, 1.4 ± 1.2, and 0.7 ± 0.5%, respectively, for the distal phalanx, proximal phalanx, and metatarsal of the big toe; 1.8 ± 1.5, 1.7 ± 1.3, 1.7 ± 1.3, and 0.6 ± 0.5%, respectively, for the distal phalanx, middle phalanx, proximal phalanx, and metatarsal of the second toe. The intraclass correlation coefficient (ICC) of the two measurements for each bone length ranged from 0.937 to 0.992. The reproducibility of these forefoot bone measurements has been reported in our previous studies^13,14^. The calculated forefoot bone lengths of the big and second toes were totaled to assess overall forefoot bone length for each toe. The total forefoot bones for each toe were normalized to the maximal foot length and used for analyses of this study.

The lengths and heights of the talus and calcaneus were measured as maximal distances between the cortexes at the distal and proximal ends. The coefficient of variations of the two measurements for the lengths and height of the talus and calcaneus in all participants were: 0.5 ± 0.4 for the talus length, 0.4 ± 0.2% for the calcaneus length, 0.6 ± 0.6 for the talus height, and 0.6 ± 0.4% for the calcaneus height. The intraclass correlation coefficient of the two measurements for the lengths and heights of each bone ranged from 0.989 to 0.996. The lengths and heights of the talus and calcaneus were normalized to the maximal foot length and body height, respectively, and these relative values were also used for analyses in this study.

### Foot arch height

MRI-measured foot arch height at non-weight bearing was measured as the minimum vertical distance between the navicular and the line connecting the thenar eminence to the bottom of the heel. The coefficient of variation of the two measurements for the foot arch height in all participants was 0.5 ± 0.4%. The intraclass correlation coefficient of the two measurements for the foot arch height was 0.997. The foot arch height was normalized to the maximal foot length (i.e., foot arch height/foot length × 100), which represented the foot arch height index^22^. The foot arch height index was also used for analyses in this study.

### Statistical analysis

The data are presented as the mean ± SD. Comparisons of measured variables between faster and slower groups of sprinters were performed using an unpaired *t*-testing. The Cohen’s *d* effect size using the pooled SD was calculated to determine the magnitudes of differences in the measured variables between the two groups. This effect size were interpreted as small (0.20–0.49), medium (0.50–0.79) and large (> 0.80)^26^. Relationships between the foot bone morphological variables and personal best 100-m sprint time in sprinters was examined using the Pearson’s product-moment correlation coefficient. The statistical significance level was defined at *P* < 0.05. All statistical analyses were conducted using IBM SPSS software (version 19.0; International Business Machines Corp, USA).

## Results

### Comparisons of foot morphological variables between faster and slower sprinters

Physical characteristics and foot morphological variables between faster and slower groups of sprinters are listed in Table 1. Physical characteristics (i.e., body height, body weight, and body mass index) did not differ significantly between the faster and slower groups. The foot length of the big toe (i.e., heel to big toe) but not the second toe was significantly higher in slower group than in faster group (*P* = 0.009), with medium effect size (*d* = 0.73). The maximum foot length was also significantly higher in slower group than in faster group (*P* = 0.017), with medium effect size (*d* = 0.66).

**Table 1:** Physical characteristics and foot morphological variables in faster and slower groups of sprinters.

	Faster sprinters (n = 28)	Slower sprinters (n = 28)	*P* value	Cohen’s *d*
Body height, cm	175.3 ± 5.2	174.4 ± 4.9	0.540	0.16
Body weight, kg	66.8 ± 5.7	64.7 ± 5.4	0.168	0.37
Body mass index, kg/m^2^	21.7 ± 1.4	21.3 ± 1.2	0.164	0.37
Heel to big toe, cm	**25.8** ± **0.9**	**26.4** ± **0.7**	**0.009**	**0.73**
Heel to second toe, cm	25.7 ± 1.0	26.1 ± 0.8	0.124	0.42
Maximum foot length, cm	**25.9** ± **0.9**	**26.4** ± **0.7**	**0.017**	**0.66**
Foot arch height, mm	**52.3** ± **5.0**	**48.1** ± **4.3**	**0.001**	**0.91**
Foot arch height index	**20.2** ± **1.8**	**18.2** ± **1.7**	** < 0.001**	**1.15**
**Forefoot bone length, mm**				
Big toe	122.5 ± 5.9	122.5 ± 5.2	0.998	0.00
Second toe	136.2 ± 6.5	135.2 ± 5.6	0.513	0.18
**Rearfoot bone length, mm**				
Talus	60.1 ± 3.6	60.3 ± 3.1	0.874	0.04
Calcaneus	80.0 ± 4.5	79.8 ± 3.0	0.812	0.06
**Rearfoot bone height, mm**				
Talus	39.6 ± 2.2	40.1 ± 2.5	0.373	0.24
Calcaneus	**55.7** ± **3.0**	**52.5** ± **2.5**	** < 0.001**	**1.20**

Values are presented as Mean ± SD. Bold fonts indicate significant difference (*P* < 0.05) between groups.

With regard to absolute lengths and heights of the foot bones, the total lengths of the forefoot bones of the big and second toes did not differ significantly between faster and slower groups of sprinters (Table 1). Moreover, the lengths of the talus and calcaneus did not differ significantly between the two groups. Furthermore, the talus height did not differ significantly between groups. In contrast, the calcaneus height was significantly higher in faster group than in slower group (*P* < 0.001), with large effect size (*d* = 1.20).

**Figure 2:**
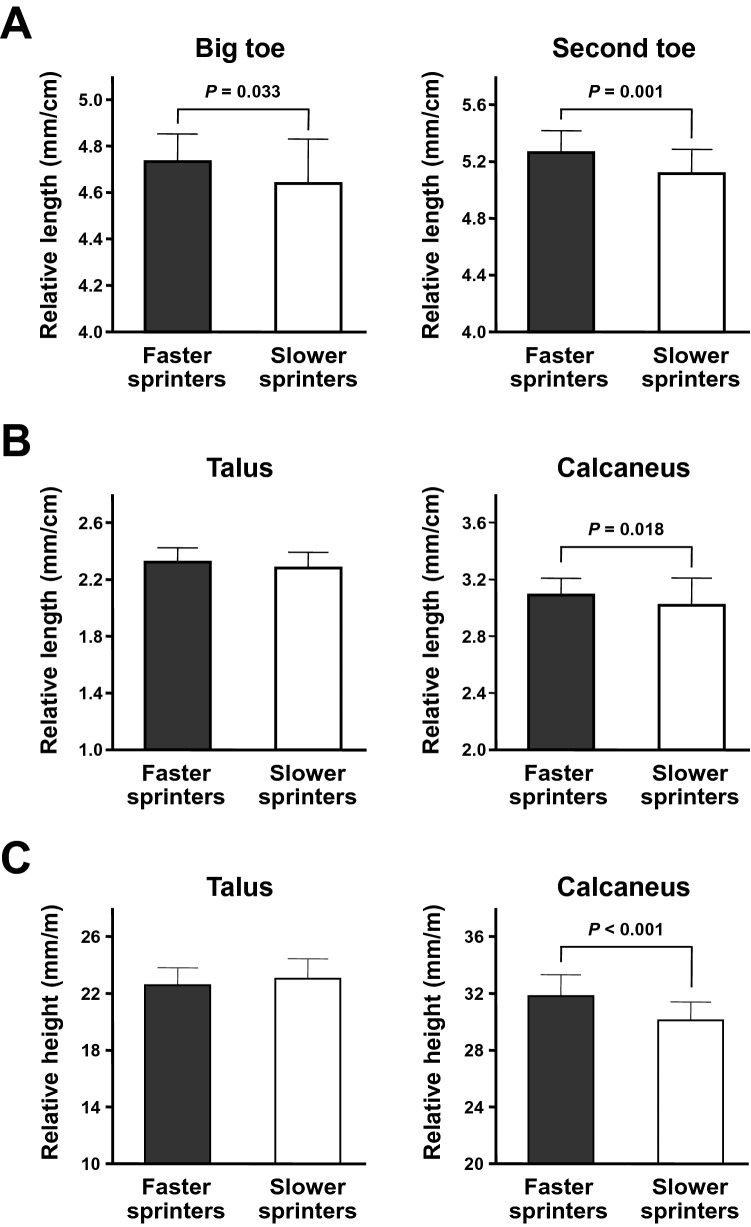
Comparisons of the relative foot bone variables between faster and slower groups of sprinters. Values are presented as Mean ± SD. Sprinters were divided into faster and slower groups (n = 28 for each) based on their personal best 100-m sprint times. The relative lengths and heights of the foot bones were calculated by normalizing to the maximal foot length and body height, respectively. (**A**) The comparisons of the relative forefoot bone lengths of the big and second toes between faster and slower sprinters. (**B**) The comparisons of the relative lengths of the talus and calcaneus between faster and slower sprinters. (**C**) The comparisons of the relative heights of the talus and calcaneus between faster and slower sprinters.

Comparisons of the relative lengths and heights of the foot bones between faster and slower groups of sprinters are presented in Fig. 2. In contrast to the absolute lengths, the relative total lengths of the forefoot bones of the big and second toes were significantly higher in faster group than in slower group (both *P*s < 0.05), with medium and large effect size (*d* = 0.59 and 0.90, respectively). Moreover, the relative length of the calcaneus but not the talus was significantly higher in faster group than in slower group (*P* = 0.018), with medium effect size (*d* = 0.65). Furthermore, the relative calcaneus height was significantly higher in faster group than in slower group (*P* < 0.001), with large effect size (*d* = 1.22).

### Relationships between foot morphological variables and sprinter performance in sprinters

**Figure 3:**
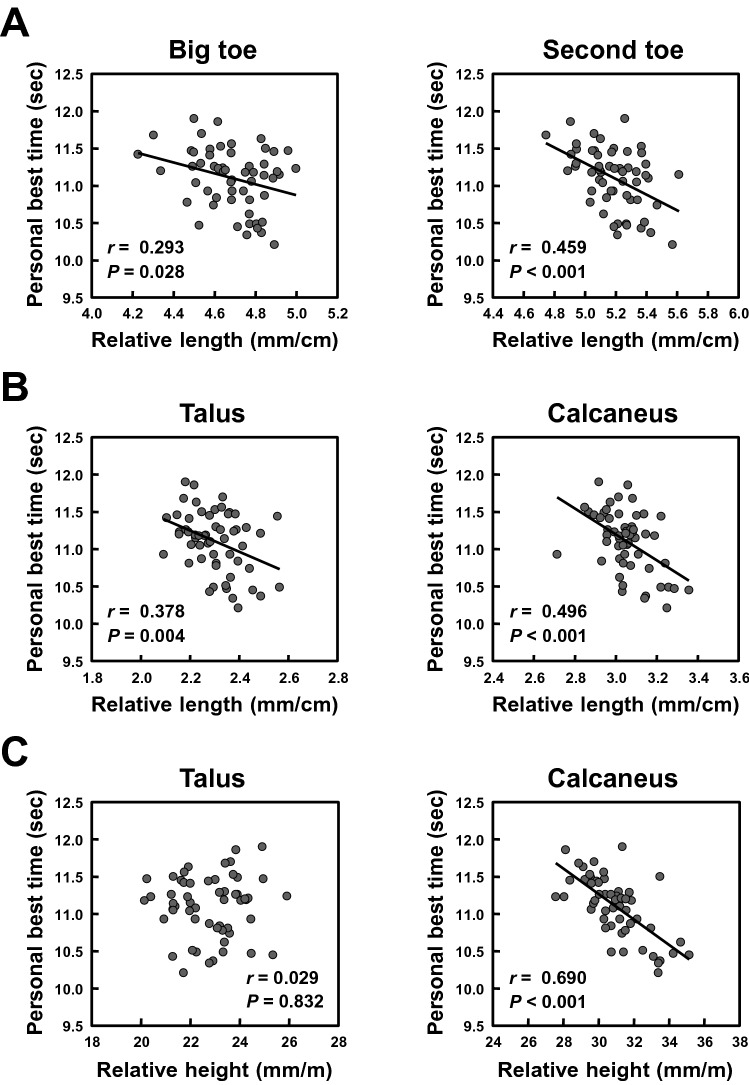
Relationships between the relative foot bone variables and sprint performance in sprinters. (**A**) The relationships between the relative forefoot bone lengths of the big and second toes and personal best 100-m sprint time. (**B**) The relationships between the relative lengths of the talus and calcaneus and personal best 100-m sprint time. (**C**) The relationships between the relative heights of the talus and calcaneus and personal best 100-m sprint time.

Relationships of the relative lengths and heights of the forefoot and rearfoot bones with sprint performance in sprinters are presented in Fig. 3. The relative total lengths of the forefoot bones of the big and second toes correlated significantly with personal best 100-m sprint time (Fig. 3A; *r* =  − 0.293 and − 0.459, both *P*s < 0.05). Similarly, the relative lengths of the talus and calcaneus correlated significantly with personal best 100-m sprint time (Fig. 3B; *r* =  − 0.378 and − 0.496, both *P*s < 0.01). There was no significant correlations between the relative talus height and personal best 100-m sprint time (Fig. 3C; *r* = 0.029, *P* = 0.832). In contrast, the relative calcaneus height correlated significantly with personal best 100-m sprint time (Fig. 3C; *r* =  − 0.690, *P* < 0.001).

Correlation coefficients between the relative rearfoot bone heights and the relative foot bone lengths in sprinters are shown in Table 2. A significant correlation was observed between the relative talus height and the relative forefoot length of the big toe (*r* =  − 0.413, *P* = 0.002). Although there was no significant correlation between the relative calcaneus height and the relative forefoot length of the big toe, the relative calcaneus height correlated significantly with the relative forefoot length of the second toe (*r* = 0.328, *P* = 0.014). Similarly, the relative calcaneus height correlated significantly with the lengths of the talus and calcaneus (*r* = 0.323 and 0.306, respectively, both *P*s < 0.05).

**Table 2:** Correlation coefficients between the relative rearfoot bone heights and the relative foot bone lengths.

	Talus height		Calcaneus height	
	*r*	*P*	*r*	*P*
Big toe length	− **0.413**	**0.002**	0.189	0.163
Second toe length	− 0.135	0.320	**0.328**	**0.014**
Talus length	0.220	0.140	**0.323**	**0.015**
Calcaneus length	0.100	0.464	**0.306**	**0.022**

Bold fonts indicate significant correlation (*P* < 0.05) between variables.

**Figure 4:**
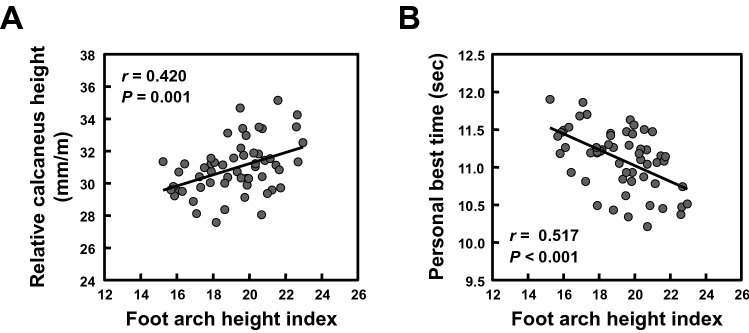
Relationships of the foot arch height index with the relative calcaneus height and sprint performance in sprinters. The foot arch height index was calculated as [foot arch height/maximal foot length × 100]. (**A**) The relationship between the foot arch height index and the relative calcaneus height. (**B**) The relationship between the foot arch height index and personal best 100-m sprint time.

Relationships of the foot arch height index with the calcaneus height and sprint performance in sprinters are presented in Fig. 4. The foot arch height and its index were significantly higher in faster group than in slower group of sprinters (Table 1; both *P*s ≤ 0.001), with large effect size (*d* = 0.91 and 1.15, respectively). Although there was no significant correlation between the talus height and foot arch height (*r* =  − 0.017, *P* = 0.903), the calcaneus height correlated significantly with the foot arch height (*r* = 0.552, *P* < 0.001). Such a significant correlation was also observed between the relative calcaneus height and foot arch height index (Fig. 4A; *r* = 0.420, *P* = 0.001). Additionally, the foot arch height correlated significantly with personal best 100-m sprint time (*r* =  − 0.460, *P* < 0.001). Similarly, a significant correlation was observed between the foot arch height index and personal best 100-m sprint time (Fig. 4B; *r* =  − 0.517, *P* < 0.001).

## Discussion

We found that the lengths of the forefoot and rearfoot bones correlated with personal best 100-m sprint time in sprinters. Moreover, the height of the calcaneus, but not that of the talus, correlated with the sprint performance. Furthermore, the calcaneus height correlated with the forefoot and rearfoot bone lengths. Additionally, the calcaneus height correlated with the foot arch height, and the foot arch height correlated with sprint performance. These findings suggest that a taller calcaneus may play an important role in modeling longer forefoot and rearfoot bones and higher foot arch height and archiving better sprint performance in sprinters.

We previously reported that longer forefoot bones correlated with better sprint performance in sprinters^13,14^; therefore, with an increase in sample size, the present finding further clarify the result of our previous studies. Using computer simulation, Lee and Piazza^12^ reported that a longer forefoot contributes to the production of greater plantar flexor torque during the push-off phase while sprinting. Additionally, van Werkhoven and Piazza^27^ determined that a longer hallux correlated with higher jump height in a single-joint jump task, possibly due to increased push-off strength. Jump performance is a major physical performance factor for predicting sprint performance in sprinters^28,29^. The findings of the present and previous studies suggest that the longer forefoot bones are advantageous morphologies for superior performance of sprinters, potentially because of increases in MTP flexor (i.e., push-off strength) and plantar flexor torques during sprinting.

Our first hypothesis was that the longer rearfoot bones would be related to superior sprint performance in sprinters. In this study, we found that the calcaneus, but not the talus, was longer in faster group than in slower group of sprinters. Furthermore, longer calcaneus and talus bones correlated with better sprint performance in sprinters. These findings suggest that the lengths of the rearfoot bones, especially the calcaneus, in addition to the forefoot bones may be related to superior sprint performance in sprinters. In a study by van Werkhoven and Piazza^27^, they reported that longer heel length, in addition to hallux length, correlated with higher single-joint jump height. Their results suggest that both longer forefoot and rearfoot bones can coexist in the foot and may additively contribute to superior sprint performance. To the best of our knowledge, the present study is the first to determine the relationship between the rearfoot bone lengths and athletic performance, including sprinting.

Our second hypothesis was that the taller calcaneus would be required for modeling the favorable foot bone morphologies (i.e., longer forefoot and rearfoot bones) and would be related to superior sprint performance. In this study, we found that the calcaneus height was higher in faster group than in slower group of sprinters. Moreover, a taller calcaneus correlated with better sprint performance in sprinters. Furthermore, the taller calcaneus correlated with the longer forefoot and rearfoot bones. These findings support our hypothesis prior to the present study. To the best of our knowledge, the present study is also the first to determine the relationship between the calcaneus height and athletic performance, including sprinting.

Our third hypothesis was that a taller calcaneus would be related to a higher foot arch height. We also hypothesized that the higher foot arch height would be related to superior sprint performance. In this study, we found that the foot arch height was higher in faster group than in slower group of sprinters. Furthermore, the higher foot arch height correlated with better sprint performance in sprinters. Previous studies reported that the foot arch height may contribute to increasing the MTP joint and plantar flexor torques during the push-off phase while sprinting^11,12,19–21^. Morita et al.^22^ reported a positive correlation between foot arch height and sprint performance in children. Despite these findings, to the best of our knowledge, no study has examined this relationship in adults, including sprinters, prior to the present study. Therefore, this is the first study to determine a positive relationship between foot arch height and sprint performance in adult sprinters. In addition, although the foot arch height is often associated with the foot length in non-sprinters^23,24^, the present study determined no correlation between foot arch height and foot length (e.g., maximal foot length) in sprinters (*r* = 0.055, *P* = 0.687). Furthermore, a trend against negative correlation was observed between maximal foot length and sprint performance (*r* = 0.235, *P* = 0.081). Therefore, a higher foot arch height may be related to better sprint performance independently of the foot length in sprinters. Taken together, we suggest that a taller calcaneus may help achieve superior sprint performance, potentially by modeling the functional foot morphology (i.e., a higher foot arch height) in addition to the longer forefoot and rearfoot bones.

The calcaneus and talus are known to be pivotal bones for foot arch formation^30,31^. The results of this study showed that although there were no correlations of absolute and relative heights of the talus with the foot arch height and its index, absolute and relative heights of the calcaneus correlated positively with the foot arch height and its index. Using radiographs Agoada and Kramer^30^ reported that in the course of weight-bearing, a taller calcaneus may be related to a higher foot arch height by showing a positive correlation of the ratio of the height relative to the length of the calcaneus with the foot arch height. In an additional analysis conducted in the present study, we similarly obtained a positive correlation of the ratio of the height to the length of the calcaneus with the foot arch height when non-weight-bearing (r = 0.294, *P* = 0.028). By contrast, in a study by Agoada and Kramer^30^, all variables, including the ratio of the height to the length, related to the height of the talus did not correlate with the foot arch height. Furthermore, the result of additional analysis in the present study showed a negative correlation of the ratio of the height to the length of the talus with the foot arch height (r =  − 0.448, *P* = 0.001), suggesting that a taller talus may be a negative morphological factor for modeling a higher foot arch height. As an explanation of these findings of the present and previous studies, the high arch of the foot is related to an increase in the calcaneal inclination angle (i.e., the angle between the inferior surface of the calcaneus and the supporting surface), which may contribute to modeling a taller calcaneus^31^. Contrary to this, the low arch of the foot is related to an increase in the calcaneal-first metatarsal angle (i.e.., the angle formed by the inferior surface of the calcaneus and a line parallel to the dorsum of the mid-shaft of the first metatarsal), which may contribute to modeling a taller talus^31^. Therefore, a taller calcaneus due to increased calcaneal inclination angle may be a positive factor for a higher foot arch height, whereas a taller talus due to increased calcaneal-first metatarsal angle may be a negative factor for a higher foot arch height. In summary, the height of the calcaneus, but not the talus, may play an important role in increasing the foot arch height.

This study has several limitations. First, although we recruited only Japanese male sprinters, it remains unclear whether the present findings can be generalized to other racial and ethnic groups or to female sprinters. Further studies are needed to determine the relationship between the foot morphological variables and sprint performance in sprinters of different races and sex. Second, although we measured the foot arch height when non-weight bearing because of a technical limitation of magnetic resonance imaging, the foot arch height at weight-bearing may be a more important parameter for predicting sprint performance than that at non-weight bearing. This is potentially due to a general form during human movements. Further studies are needed to determine the relationship between the foot arch height when weight-bearing and sprint performance. Third, although we determined that the foot bone morphologies were different between faster and slower groups of sprinters based on personal best 100-m sprint time, it is unclear whether these differences are derived from genetic and/or long-term training effects. Nevertheless, although the forefoot bones may be determined mainly by genetic factors^32^, the calcaneus may be more sensitive to acquired factors (e.g., long-term sprint training) than other foot bones such as the forefoot bones^33^. Based on the findings of the present and previous studies, successful sprinters are characterized by greater calcaneus, whereas successful endurance runners may be characterized by smaller calcaneus^15,34,35^, suggesting that the calcaneus morphology may be favorably modeled throughout long-term specific training. Further longitudinal studies are needed to follow-up the growth and development of the foot bones in junior athletes, including sprinters and endurance runners.

In a perspective, it would be generally difficult to remodel the length and height of the foot bones in adult humans. Nevertheless, an increase in calcaneal inclination angle may contribute to increasing the calcaneus height based on an analysis method of this study; this implies that the calcaneal inclination angle may have potential as an important morphological parameter of sprint performance in sprinters. Based on a positive correlation between calcaneal inclination angle and foot arch height^31^, an increase in the foot arch height may become the important target of regular training for enhancing sprint performance in sprinters. This is because the foot arch height can be increased by short foot exercise training^36–38^. Mulligan and Cook^37^ reported that the short foot exercise training increased the foot arch height when weight bearing in healthy young adults. Therefore, further studies are needed to examine the effect of short foot exercise training on the foot arch height and sprint performance in sprinters.

In conclusion, we suggest that the taller calcaneus may be a key morphological factor for achieving superior sprint performance, potentially via modeling the longer forefoot and rearfoot bones and higher foot arch height. These findings could enhance our understanding of the importance of the foot morphology on athletic performance in athletes.

## Data availability

The datasets used for this study are available from the corresponding author on reasonable request.
